# Sustainability of programs to reach high risk and marginalized populations living with HIV in resource limited settings: implications for HIV treatment and prevention

**DOI:** 10.1186/1471-2458-11-701

**Published:** 2011-09-14

**Authors:** Brian T Montague, Bea Vuylsteke, Anne Buvé

**Affiliations:** 1 Warren Alpert School of Medicine at Brown University, Miriam Hospital, 164 Summit Ave, Providence, RI 02906, USA; 2 Department of Public Health and ITM HIV/AIDS Center, Institute of Tropical Medicine, Nationalestraat 155, B-2000 Antwerp, Belgium

## Abstract

The experiences of the past 10 years have shown that it is feasible to treat HIV infected patients with ART even in severely resource constrained settings. Achieving the levels of antiretroviral coverage necessary to impact the course of the HIV epidemic remains a challenge and antiretroviral therapy coverage in most nations remains short of even current recommendations. Though treatment as prevention and seek, test, treat and retain strategies are attractive, realization of the benefits of these strategies will require the ability to successfully engage key hard to reach populations such as sex workers. The successes engaging these populations in research settings as seen in the article by Huet et al are encouraging, however key questions remain regarding the sustainability of these efforts as patients are transitioned back to national HIV control programs, many of which are struggling even to maintain the current panels in care in the face declining external funding for HIV care. To achieve the critical goals of increasing treatment uptake and retention and thereby curtail the epidemic of HIV, advocacy from both medicine and public health providers will be critical to generate the support and political will necessary to sustain and enhance the necessary HIV care programs worldwide.

## 

When the first programs providing access to antiretroviral treatment were launched in Africa in the early years of the 21^st ^century, they were met with a lot of skepticism. There were concerns about the costs and the feasibility, and about adherence. The experiences of the past 10 years have shown that it is feasible to treat HIV infected patients with ART even in severely resource constrained settings, and that immunological reconstitution and virological suppression can be achieved for large numbers of patients resulting in declines in mortality [1]. Nonetheless there are still huge challenges ahead. In surveillance data from the World Health Organization in 2009, antiretroviral coverage according to 2006 guidelines with a CD4 cell count threshold of < = 200 was 52% worldwide. Coverage based on the higher 2010 threshold of CD4 count < = 350 was only 36%. Only 8 low or middle income countries have been able to achieve ART coverage rates of greater than or equal to 80% [2]. A substantial portion of HIV care has been subsidized through contributions from programs such as the US President's Emergency Plan for AIDS Relief (PEPFAR) and the Global Fund and it is unclear whether the present level of funding will be maintained, let alone increased.

In the context of limited and at times declining funding for antiretroviral therapy (ART), ART programs targeting marginalized and difficult to reach populations such as female sex workers (FSW), may not be considered a priority. Concerns about feasibility and adherence in ART programs for FSW resonate with the skepticism that was voiced 10 years ago about ART programs in low resource settings. ART programs for FSW indeed do face a number of challenges. FSW are hard to reach and tend to be mobile; and adhering to a strict treatment regimen may be difficult for them because of the nature of their work and their lifestyles. The question is whether these barriers can be overcome. The study by Huet et al. demonstrates that it is possible to achieve high levels of immunological reconstitution and virological suppression among FSW, and that treatment with ART of FSW can give results that are nearly comparable to those obtained amongst women in the general population.

However, as Huet et al themselves point out, their results were obtained within the framework of a research study which used frequent visits combined with intensive counseling and case management services to maintain patients in care. There are several programs in sub-Saharan Africa that offer prevention and care services to FSW with the aim to reduce the transmission of HIV from FSW to their male clients and partners. Some of these programs have started providing ART to their clients or have set up referral systems. So far, no data have been published on the levels of adherence and virological suppression achieved amongst FSW in these programs, but the results will likely be less successful than those presented by Huet et al. Moreover, these results from Burkina Faso cannot be readily extrapolated to other settings in Africa. For instance, alcohol abuse may be more common in FSW in other parts of Africa, especially Eastern and Southern Africa, and interfere with adherence [3,4]. The challenge will be the development of affordable and scalable programs to optimize adherence. Not offering ART to FSW is not an option. The recent release of results from the HPTN 052 trial confirming the efficacy of antiretroviral therapy in the prevention of HIV transmission highlights the potential importance of ART programs as part of comprehensive efforts to reduce HIV transmission, including transmission from FSW to their male clients and partners [5].

The sustainability of HIV prevention programs targeting FSW has been a challenge ever since the late 1980's when these programs were first piloted. This challenge will become even greater when care with ART is integrated into the programs. To be sustained, the interventions of necessity must be transferred to the national HIV care program. National HIV program managers, particularly in the era of declining funding through programs such as PEPFAR, are put in the position of balancing high cost case management interventions against expanded access to antiretroviral medications for the broader population. In this context, what appears to be a sustainable intervention when subsidized by outside grant funding becomes difficult to maintain over the long-term.

The Seek, Test, Treat and Retain strategy offers an attractive model over the long run for controlling the HIV epidemic. This article, while providing confirmation that even the hardest to reach populations can be reached with enough resources, highlights the challenges that will be faced in realizing the potential benefits of this strategy. Though expanded access to treatment for the 75% of the population may be feasible within the structures of national HIV care programs, reaching the hard to reach populations and sustaining the adherence to ARV treatment that will be necessary to truly curb the epidemic will require substantial resources over and above the cost of provision and monitoring of antiretroviral therapy. To achieve these goals and curtail the epidemic of HIV, advocacy from both medicine and public health providers will be critical to generate the support and political will necessary to sustain and enhance HIV care programs worldwide.

## Competing interests

The authors declare that they have no competing interests.

## Pre-publication history

The pre-publication history for this paper can be accessed here:

http://www.biomedcentral.com/1471-2458/11/701/prepub
